# Supplementary material for: Comparison of three short-course rifamycin-based regimens for the prevention of tuberculosis in patients with end-stage kidney disease: Study protocol for a randomised clinical trial (RIFAKiD-TB trial)
Source: PLoS One. 2022 Oct 21;17(10):e0276387. doi: 10.1371/journal.pone.0276387 (PMC9586383; doi:10.1371/journal.pone.0276387)
Supplement: S1 File — (DOCX) [file pone.0276387.s001.docx]

The RIFAKiD-TB Trial

COMPARISON OF TREATMENT COMPLETION RATES WITH THREE SHORT-COURSE RIFAMYCIN-BASED REGIMENS (three months of daily isoniazid plus rifampicin [3HR], three months of once-weekly isoniazid plus rifapentine [3HP], AND four months of daily rifampicin [4R]) FOR TREATMENT OF LATENT TUBERCULOSIS INFECTION IN PATIENTS WITH END-STAGE KIDNEY DISEASE: A RANDOMISED CONTROLLED CLINICAL TRIAL

**Comparison of treatment completion rates with three short-course rifamycin-based regimens (three months of daily isoniazid plus rifampicin [3HR], three months of once-weekly isoniazid plus rifapentine [3HP], and four months of daily rifampicin [4R]) for treatment of latent tuberculosis infection in patients with end-stage kidney disease: A randomised controlled clinical trial**

**(RIFAKiD-TB Trial)**

**Protocol identifying number**

**EUDRACT number:** 2021-003995-15

**Sponsor protocol identification code:** PI21/004444

**CinicalTrials.gov identifier:** NCT05021731

**Version:** 1.0

**Date:** 01.03.2022

**Sponsor:**

**Miguel Santin, MD, PhD**

**Service of Infectious Diseases**

**Bellvitge University Hospital-Bellvitge Institute for Biomedical Research**

**INFORMATION OF THE STUDY**

- **Title:**

Comparison of treatment completion rates with three short-course rifamycin-based regimens (three months of daily isoniazid plus rifampicin [3HR], three months of once-weekly isoniazid plus rifapentine [3HP], and four months of daily rifampicin [4R]) for treatment of latent tuberculosis infection in patients with end-stage kidney disease: A randomised controlled clinical trial (RIFAKiD-TB Trial)

- **Sponsor’s code:** PI21/004444
- **EudraCT number:** 2021-003995-15
- **Date:** 1 of September 2021
- **Sponsor:**

Miguel Santin

Tuberculosis Unit, Service of Infectious Diseases

Bellvitge University Hospital-Bellvitge Institute for Biomedical Research (IDIBELL)

Av/ Feixa Llarga s/n. 08907 L'Hospitalet de Llobregat, Barcelona

Phone number: +34 932 607 456

- **CRO (Monitor and pharmacovigilance):**

IDIBELL Clinical Research and Clinical Trials Unit (UICEC IDIBELL)

e-mail: [uicec@idibell.cat](mailto:uicec@idibell.cat)

Phone number: +34 932 607 107

- **Reference Ethics Committee:**

CEIm Bellvitge University Hospital

Av/Feixa Llarga s/n. 08907 L’Hospitalet de Llobregat, Barcelona

- **Trial investigators:**

1. **Bellvitge University Hospital-IDIBELL**

Principal investigator and coordinator:

Miguel Santin

Tuberculosis Unit, Service of Infectious Diseases

Bellvitge University Hospital-Bellvitge Institute for Biomedical Research (IDIBELL)

Av/ Feixa Llarga s/n. 08907 L'Hospitalet de Llobregat. Barcelona

Phone: +34 932 607 625

1. **Hospital Universitari Valle Hebrón**

Principal investigator:

Maria L. de Souza Galvao

Tuberculosis Unit

Hospital Universitari Valle Hebrón-Drassanes

Carrer Sant Oleguer, 17, 08001, Barcelona

Phone: +34 934 893 000

1. **Complexo Hospitalario de Pontevedra**

Principal investigator:

Luis Anibarro García

Infectious Diseases Unit, Service of Internal Medicine

Complexo Hospitalario de Pontevedra

C/Loureiro Crespo nº 2, 36001 Pontevedra

Phone: +34 986 800 000

1. **Hospital General Universitario Gregorio Marañón**

Principal investigator:

Paloma Gijón Vidaurreta

Service of Clinical Microbiology and Infectious Diseases

Hospital General Universitario Gregorio Marañón

C/ Doctor Esquerdo, 46. 28007 Madrid

Phone: +34 915 868 454

1. **Hospital del Mar-Parc de Salut Mar**

Principal investigator:

Francesca Sánchez Martínez

Tuberculosis Unit, Service of Infectious Diseases

Hospital del Mar-Parc de Salut Mar

Passeig Marítim, 25-29, 08003, Barcelona

Phone: +34 932 483 000

1. **Hospital Universitario de Jerez de la Frontera**

Principal investigator:

José M. Barcala Salido

Service of Internal Medicine

Hospital Universitario de Jerez de la Frontera, Cádiz

Calle de Managua 28, 11407, Jerez de la Frontera

Phone: +34 956 032 000

1. **Hospital Universitario Virgen del Rocío**

Principal investigator:

Rafael Luque Márquez

Service of Infectious Diseases

Hospital Universitario Virgen del Rocío

Av. Manuel Siurot, s/n, 41013 Sevilla

Phone: +34 955 012 000

- **Noun and description of the drugs used in the trial:**

1. **Isoniazid**: hydrazide of isonicotinic acid, which is one of the first-line drugs for the treatment of active and latent tuberculosis (see Technical Data Sheet in Attachment 1a)
2. **Rifampicin** (or Rifampin): a semi-synthetic rifamycin B derivative, active against mycobacteria, most gram-positive bacteria, and some gram-negative bacteria (See Technical Data Sheet in Attachment 1b).
3. **Rifapentine**: is a long-acting, cyclopentyl-substituted derivative of rifamycin used to treat mycobacterial infections. It has a role as an antitubercular agent and a leprostatic drug (See Technical Data Sheet in Attachment 1c).

**Table of contents**

**1. RATIONALE AND JUSTIFICATION OF THE STUDY ……………………………………………………………8**

**2. OBJECTIVES AND PURPOSE OF THE TRIAL…………………………………………………………………… 12**

**3. TRIAL DESIGN ……………………………………………………………………………………………………………. 12**

**3.1. Statement of the endpoints…………………………………………………………………………12**

**3.2. Trial type……………………………………………………………………………………………………..12**

**3.3. Randomization…………………………………………………………………………………………….13**

**3.4. Description of the treatment………………………………………………………………………..13**

**3.5. Data recorded from the participants…………………………………………………………….14**

**3.6. End of the trial…………………………………………………………………………………………..….14**

**4. SELECTION AND WITHDRAWAL OF PARTICIPANTS…………………………………………………………. 14**

**4.1. Inclusion criteria…………………………………………………………………….………………………..14**

**4.2. Exclusion criteria……………………………………………………………………………………………..15**

**4.3. Participant withdrawal criteria………………………………………………………………………..15**

**4.4. Follow-up of participants withdrawn from the trial………………………………………….15**

**5. TRIAL INTERVENTION…………………………………………………………………………………………………….. 16**

**5.1. Description of the intervention…………………………………………………..……………………16**

**5.2. Drugs: storage and administration…………………………………………………………………17**

**5.3. Medications permitted and not permitted during the trial……………………………..18**

**5.4. Discontinuation of the study drugs due to adverse events………………………………19**

**6. ASSESSMENT OF EFFICACY………………………………………………………………………………………………20**

**6.1. Efficacy parameters………………………………………………………………………………………..20**

**6.2. Methods and timing for assessment……………………………………………………………….20**

**7. ASSESSMENT OF SAFETY …………………………………………………………………………………………………20**

**7.1. Monitoring, recording, and reporting of adverse events………………………………….20**

**7.2. Reference safety information…………………………………………….…………………………….21**

**7.3. Evaluation of adverse events…………………………………………………………………………..21**

**7.4. Abnormal laboratory values…………………………………………………………………………….23**

**7.5. Pregnancy………………………………………………………………………………………………………..24**

**7.6. Reporting of serious adverse events………………………………………………………………..25**

**7.7. Procedure for expedited reporting of unexpected adverse events……………………25**

**7.8. Special interest adverse events………………………………………………………………………..26**

**7.9. Annual safety report………………………………………………………………………………………..26**

**7.10. Report to the investigators…………………………………………………………………………….26**

**7.11. Data and safety monitoring board (DSMB)…………………………………………………….27**

**8. STATISTICAL METHODS…………………………………………………………………………………………………..27**

**8.1. Statistical analysis……………………………………………………………………………………………27**

**8.2. Sample size………………………………………………………………………………………………………28**

**9. ETHICS…………………………………………………………………………………………………………………………….28**

**9.1. Informed consent…………………………………………………………………………………………….28**

**9.2. Subjects’ confidentiality…………………………………………………………………………………..29**

**10. FUNDING AND INSURANCE……………………………………………………………………………………………29**

**11. AGREEMENT TO PUBLISH………………………………………………………………………………………………29**

**12. ATTACHMENTS……………………………………………………………………………………………………………..29**

**1. RATIONALE AND JUSTIFICATION OF THE STUDY**

The World Health Organization (WHO) regards the diagnosis and treatment of latent tuberculosis (TB) infection as an essential intervention to achieve the goal of the End TB Strategy by 2035 and the subsequent elimination by 2050 (1). Screening for and treatment of latent TB infection has become a priority as the pool of people at risk increases due to life expectancy improvement, along with chronic underlying diseases and immunosuppression conditions.

The end stage kidney disease (ESKD) increases by a factor of 6 to 25 the risk of TB, and the prevalence of TB among recipients of kidney transplant is 20- to 74-fold higher than in the general population (2). Available evidence, albeit scarce, supports systematic screening and treatment of these patients, and it is recommended by the guidelines (3, 4). The recommended treatment includes isoniazid (INH) for 6-9 months (6-9H), rifampicin (RMP) and INH combination for 3 months (3HR), or RMP alone for 4 months (4R).

The ESKD patients constitute a fragile population, with frequent intercurrent clinical events, polypharmacy, which often make latent TB difficult to treat, mainly because of adverse events. Data from the TB Unit of the Bellvitge University Hospital with 387 ESKD patients treated with 3HR, 6-9H or 4R prior kidney transplant, showed that 88% completed treatment satisfactorily, and adverse events were the commonest cause of permanent discontinuation (5). Central nervous system toxicity caused by INH is of particular concern in these patients (6-8). Seizures, encephalopathy, dizziness, and cerebellar syndrome have been reported. In our own experience, 9% of patients treated with regimens including INH presented dizziness, referred to as feeling “drunk”. In all the cases, it was invalidating, and improved in a few days after discontinuing treatment. Neurotoxicity has been related to the inhibition of the pyridoxin phosphorylation to the active metabolite, the pyridoxal 5-phosphate. In patients with ESKD, and particularly those under substitutive renal therapy, accumulation of INH metabolites along with the pyridoxal 5-phosphate removal by dialysis, lead to neurotoxicity. In addition, polymorphisms of the N-acetyltransferase (NAT)-2 *2, *5, *6, *7 and *14 alleles, which confer the slow- acetylator phenotype, would contribute to increasing levels of INH and its metabolites, and risk of neurotoxicity (9).

The short-course rifampicin-containing regimens, with or without INH, are currently recommended as the first line treatment for latent TB infection. The combination of weekly rifapentine (RPT) and INH, under supervised administration for 3 months (3HP), has been shown to be no-inferior to the 9H regimen (10), and has already been approved for the first-line treatment of latent TB infection in the USA (11). A metanalysis showed that 87.5% of subjects treated with the 3HP regimen completed treatment, as compared with 65.9% of those treated with INH- or RMP-containing regimens (12). However, the available evidence in ESKD patients is limited to a small number of cases reported in the literature (13-15). A retrospective study with 153 candidates to kidney transplant with latent TB, 93% of the 43 3HP-treated patients and 47% among the 110 treated with the INH for 9 months completed treatment (13). In another two studies, including 17 and 12 transplant candidates treated with 3HP, completion rates were 76% and 100% respectively (14, 15).

The unanimity of the guidelines about the systematic treatment of latent TB in patients with ESKD contrasts to the limited evidence on safety, tolerability, and completion rates of treatment in this population. Among other reasons, ESKD patients and candidates to kidney transplant have been systematically excluded from clinical trials evaluating latent TB treatment. Clearly, knowledge on which treatment regimens are better tolerated and more convenient for latent TB in ESKD patients is urgently needed. Precise guidance on posology and administration (dose adjustment, pre- or post-dialysis administration), toxicity (toxicity profile, how to prevent it and how to monitor it), and graded alternatives are currently lacking. In line with our previous research on the optimization of diagnosis and treatment of latent TB, we aimed at assessing treatment completion of 3 short-course regimens (3HR, 4R and 3HP) for treatment of latent TB in ESKD patients. For this purpose, and based on our pre-transplant program’s data at the TB Unit of the Bellvitge University Hospital, we will compare 4R and 3HP regimens with the 3HR (control) regimen.

We will work with the hypothesis that the 3HP and 4R regimens will be better tolerated than the 3HR regimen, which will result in a better treatment completion. After an exhaustive review of the literature and the Clinical Trials Registry (https://www.clinicaltrials.gov/), we did not find any study in the field, published or registered.

**References**

1. Uplekar M, Weil D, Lonnroth K, Jaramillo E, et al. WHO's new End TB Strategy. Lancet 2015;385: 1799-1801.

2. Krishnamoorthy S, Kumaresan N, Zumla A. Latent tuberculosis infection and renal transplantation. Diagnosis and management. Int J Infect Dis. 2019;80S: S73-S76.

3. Muñoz L, Santin M. Prevention and management of tuberculosis in transplant recipients: from guidelines to clinical practice. Transplantation 2016;100: 1840-1852.

4. Currie AC, Knight SR, Morris PJ. Tuberculosis in Renal Transplant Recipients: The Evidence for Prophylaxis. Transplantation 2010;90: 695 -704.

5. Grijota-Camino MD, Montero N, Luque MJ, Díaz-Jurado M, Sabé N, Pérez-Recio, et al. Tuberculosis prevention in patients undergoing kidney transplantation: A nurse-led program for screening and treatment. Transpl Infect Dis 2021: e13603.

6. Low JM, Wong KW. Isoniazid-induced encephalopathy in an end-stage renal disease patient. A case report and literature review. Med J Malaysia 2019;74: 553-554.

7. Wang HY, Chien CC, Chen YM, et al. Encephalopathy caused by isoniazid in a patient with end stage renal disease with extrapulmonary tuberculosis. Renal Failure 2003;25: 135-138.

8. Bhowmik D, Mahapatra HS, Mhajan S., et al. Isoniazid induced acute bilateral cerebellar syndrome in chronic kidney disease. Clin Nephrol 2007;67: 63-64.

9. Constantinescu SM, Buysschaert B, Haufroid V, et al. Chronic dialysis, NAT2 polymorphisms, and the risk of isoniazid-induced encephalopathy. Case report and literature review. BMC Nephrology 2017;18: 282.

10. Sterling TR, Villarino ME, Borisov AS. Three Months of Rifapentine and Isoniazid for Latent Tuberculosis Infection. N Engl J Med 2011;365: 2155 -2166.

11. Sterling TR, Njie G, Zenner D, Cohn DL, Reves R, Ahmed A, et al. Guidelines for the treatment of latent tuberculosis infection: Recommendations from the National Tuberculosis Controllers Association and CDC, 2020. MMWR Recomm Rep 2020;69(RR-1):1-11.

12. Njie GJ, Morris AB, Woodrull RY, et al. Isoniazid-Rifapentine for Latent Tuberculosis Infection: A Systematic Review and Meta-analysis. Am J Prev Med 2018;55: 244 -252.

13. Simkins J, Abbo LM, Camargo JF, et al. Twelve-Week Rifapentine Plus Isoniazid versus 9-Month Isoniazid for the Treatment of Latent Tuberculosis in Renal Transplant Candidates. Transplantation 2017;101: 1468 - 1472.

14. de Castilla DL, Rakita RM, Spitters CE, et al. Short-course isoniazid plus rifapentine directly observed therapy for latent tuberculosis in solid-organ transplant candidates. Transplantation 2014;97: 206 -211.

15. Knoll BM, Nog R, Wu Y. Three months of weekly rifapentine plus isoniazid for latent tuberculosis treatment in solid organ transplant candidates. Infection 2017;45: 335 -339.

**2. OBJECTIVES AND PURPOSE OF THE TRIAL**

- **Primary objective**

To determine if treatment completion with either three months of once-weekly isoniazid plus rifapentine (3HP) or four months of daily rifampicin (4R) or both is better in comparison to three months of daily isoniazid plus rifampicin (3HR) for treatment of latent TB infection in patients with end-stage kidney disease.

- **Secondary objective**

To determine the safety and tolerability of a 3HR, 3HP, and 4R regimens for the treatment of latent TB infection in patients with end-stage kidney disease.

**3. TRIAL DESIGN**

**3.1. Statement of the endpoints**

- **Primary endpoint**

The primary endpoint will be the proportion of participants who complete the assigned treatment regimen, defined as: 1) 90 doses within a maximum of 16 weeks, without interruptions longer than 2 weeks, and no more than on 2 occasions, for the 3HR arm (control); 2) 12 doses within a maximum of 14 weeks, without interruptions longer than 10 days, for the 3HP arm (experimental 1), and 3) 120 doses within a maximum of 20 weeks, without interruptions longer than 2 weeks, and no more than on two occasions, for the 4R arm (experimental 2).

- **Secondary endpoints**
- Proportion of participants who definitively discontinue the assigned treatment because of adverse events, regardless of its relationship to the treatment of the study.
- Proportion of participants who definitively discontinue the assigned treatment because of adverse events related to the treatment of the study.
- Crude mortality: Number of participants who die while on the study, regardless of its relationship to investigational medicinal products (IMPs) of the study.

**3.2. Trial type**

Prospective, open-label, randomized (1:1:1), superiority, multicentre clinical trial with parallel group design, comparing three short-course rifamycin-based regimens for the treatment of latent TB infection in patients with end-stage kidney disease.

**3.3. Randomization**

Eligible patients who fulfil the inclusion criteria will be randomly assigned to one of the three study arms. Randomization will be stratified for sites and for 3 subgroups according to the renal substitutive therapy (non-dialysis, on haemodialysis, or on peritoneal dialysis), in a 1:1:1 allocation ratio, using a computer-generated randomization list integrated into the Research Electronic Data Capture (REDCap) platform.

- **3HR arm (control).** Consists of three months of daily isoniazid (5 mg/Kg) plus rifampin (10 mg/Kg).
- **3HP arm (experimental 1).** Consists of three months of once-weekly isoniazid (15 mg/Kg) plus rifapentine (900 mg for ≥50 Kgs or 750 mg for 32 to 50 Kgs).
- **4R arm (experimental 2).** Consists of four months of rifampicin (10 mg/Kg).

**3.4. Description of the treatment** (for detailed information, see **Section 5** **“TRIAL INTERVENTION” section**)

- **Isoniazid**

Isoniazid is the hydrazide of isonicotinic acid and is one of the primary drugs for tuberculosis treatment. The activity of isoniazid is limited to the mycobacteria of the *M. tuberculosis* complex. It is bactericidal for rapidly dividing organisms and bacteriostatic for “resting” bacilli. The mechanism of action is the inhibition of the biosynthesis of mycolic acids, a component of the mycobacterial cell wall. It is approved in Spain for the treatment of active and latent TB.

Isoniazid will be part of two arms of the study: in combination with rifampicin (control arm) and combined with rifapentine (experimental 1 arm). In the control arm, participants will receive 5 mg/Kg (up to 300 mg) of isoniazid, administered daily, for three months (90 doses). In the experimental 1 arm, participants will receive isoniazid 900 mg weekly, combined with rifapentine, for 12 weeks.

- **Rifampicin**

Rifampicin is a semi-synthetic rifamycin derivative that is highly active against mycobacteria, most gram-positive bacteria, and some gram-negative bacteria. It is bactericidal for both intracellular and extracellular microorganisms. By inhibiting prokaryotic DNA-dependent RNA polymerase, it suppresses the early elongation of the nucleotide chain in RNA synthesis.

Rifampicin will be part of two arms of the study: in combination with isoniazid (control arm) and as monotherapy (experimental 2 arm). In the control arm, participants will receive 10 mg/Kg (up to 600 mg) of rifampicin, administered daily, for three months. In the experimental 2 arm, participants will receive 10 mg/Kg (up to 600 mg) of rifampicin, administered daily, for four months.

- **Rifapentine**

Rifapentine is a long-acting, cyclopentyl-substituted derivative of rifamycin used to treat mycobacterium infections. It has a role as an antitubercular agent and a leprostatic drug.

Rifapentine will be administered in one of the three study arms (experimental 1 arm). Participants will receive 15 mg/Kg (up to 900 mg) of rifapentine weekly, for 12 weeks, in combination with isoniazid 900 mg weekly, for 12 weeks.

**3.5. Data recorded from participants**

The master file will contain each of the documents required in the guidelines for Good Clinical Practice (CPMH/ICH/135/95). The trial staff will ensure that the participants’ anonymity is maintained. The participants will be identified only by a participant ID number on the Case Report Form (CRF) and any electronic database. All documents will be stored securely and only accessible by trial staff and authorized personnel.

CRF will include demographic data and medical history. If needed be see attachment 2 “Schedule of events” for the complementary tests needed.

**3.6. End of the trial**

The end of the trial is defined as the date of the last visit of the last participant.

**4. SELECTION AND WITHDRAWAL OF PARTICIPANTS**

**4.1. Inclusion criteria**

- Patients 18 years or older with kidney disease stage 5 (glomerular filtrate rate <15 mL/minute or under substitutive renal therapy) who require treatment for latent tuberculosis infection
- Male, or female with negative pregnancy test prior to enrolment
- Female of childbearing age willing to take appropriate barrier contraceptive measures or to abstain from heterosexual intercourse during the study therapy
- Informed written consent

**4.2. Exclusion criteria**

- Prior allergy/intolerance to rifamycins or isoniazid
- Pregnancy or breastfeeding
- Pre-treatment transaminases (ALT and/or AST) >5-fold of the upper limit of the normality
- Concomitant treatment with drugs contraindicated with the study medications
- Having received rifamycins or isoniazid within the two previous weeks
- Weigh <32 Kgs
- Inability to understand the nature of the study or to give written consent

**4.3. Participant withdrawal criteria**

Each participant has the right to withdraw study at any time. In addition, the investigator may discontinue a participant from the study at any time if they consider it necessary for any reason including:

- An adverse event that requires discontinuation of the study medication, mainly liver toxicity caused by isoniazid or rifamycins.
- Non-attendance at two consecutive visits, if it was not because of death.
- Not completing the assigned treatment, for any reason, within the scheduled frame-time (1. not completion of 90 doses within a maximum of 16 weeks, without interruptions longer than 2 weeks, and no more than on 2 occasions, for the 3HR arm [control]; 2. not completing 12 doses within a maximum of 14 weeks, without interruptions longer than 10 days, for the 3HP arm [experimental 1] , and 3. Not completion of 120 doses within a maximum of 20 weeks, without interruptions longer than 2 weeks, and no more than on two occasions, for the 4R arm [experimental arm 2]).

**4.4. Follow-up of participants withdrawn from the trial**

Participants withdrawn from the study because of adverse events will be followed up for four weeks beyond the withdrawal.

**5. TRIAL INTERVENTION**

**5.1. Description of the intervention**

- **Treatment of latent TB infection in the three study arms**
- **3HR arm (control).** Treatment will consist of daily, self-administered, 5 mg/Kg (up to 300 mg) of isoniazid plus daily 10 mg/Kg of rifampin (up to 600 mg), for three months.

Participants will receive one tablet of Cemidon® (isoniazid 300 mg/pyridoxine hydrochloride 50 mg) *(Attachment 1a)*, given on an empty stomach, plus two tablets of Rifaldin® (rifampicin 300 mg) *(Attachment 1b)* or Rimactán® (rifampicin 300 mg) *(Attachment 1c)*, given on an empty stomach. In patients under renal replacement therapy with haemodialysis, Cemidon® and Rifaldin®/ Rimactán® will be administered within two hours after finishing the dialysis session, and at the same hour the non-dialysis days. The total amount of doses taken per patient will be 90.

- **3HP arm (experimental 1).** Treatment will consist of once weekly, self-administered with phone call reminder, 15 mg/Kg (up to 900 mg) of isoniazid, plus rifapentine (900 mg for ≥50 Kgs or 750 mg for 32 to 50 Kgs), for three months.

Participants will receive three once-weekly tablets of Cemidon® (isoniazid 300 mg/pyridoxine hydrochloride 50 mg), given on an empty stomach, plus six once-weekly tablets of Priftin® (rifapentine 150 mg) *(Attachment 1d)*, given with meals. In patients under renal replacement therapy with haemodialysis, both Cemidon® and Priftin® will be administered within two hours after finishing the dialysis session, and at the same hour the non-dialysis days. The total amount of doses taken per patient will be 12.

- **4R arm (experimental 2).** Treatment will consist of self-administered daily 10 mg/Kg (up to 600 mg) of rifampicin, for four months.

Participants will receive two once-daily tablets of Rifaldin® (rifampicin 300 mg) or Rimactán® (rifampicin 300 mg), on an empty stomach. The total amount of doses taken per patient will be 120.

- **Participant’s assessment and follow-up visits**

Listed below are protocol-specified study visits for all recruited subjects *(see attachment 2 for detailed information)*:

- **Enrolment visit.** It will take place within two weeks before starting the study treatment. At this visit, patients will undergo a complete clinical assessment and review of the treatment. Active TB will have already been ruled out by clinical and radiographic assessment.

A blood sample will be obtained in a subset of 100 participants at the coordinator site (Bellvitge University Hospital), for a sub-study on polymorphisms of the N-acetyltransferase (NAT)-2, to determine the acetylator profile (slow/rapid).

- **Allocation visit.** It will be the day 0 visit. Once the written informed consent has been signed, the investigator will proceed to randomize the participant.
- **On-treatment visits.** Participants will undergo follow-up visits while on treatment at days 15, 30, 60 and 90 for the 3HR (control) and the 3HP (experimental 1) arms, and 15, 30, 60, 90 and 120 for the 4R (experimental 2) arm. Adverse events, concomitant medication, and adherence will be assessed by means of a clinical interview at each visit, and blood tests at days 30, 60 and 90 for 3HR (control) and 3HP (experimental 1) arms, or 15, 60 and 120 for the 4R (experimental 2) arm.
- **Close-out visit.** In all the three arms, participants will be assessed 4 weeks after finishing the assigned treatment, at day 120 for the 3HR (control) and 3HP (experimental 1) arms, and day 150 for the 4R (experimental 2) arm.

Visits will be face-to-face at days 30, 60 and 90 for 3HR (control) and 3HP (experimental 1) arms, or 30, 60 and 120 for the 4R (experimental 2) arm. For the 4R arm, the visit at day 90 will be also performed by a phone call. Fifteen-day and post-treatment visits will be done by means of video- or telephone-call, whenever the investigator deems it is safe, and the participant agrees.

- **Monitoring treatment compliance**

Compliance with treatment will be monitored by clinical interview, counting leftover tablets. Assessment of urine colour (orange colour caused by rifampicin) will be carried out on participants with residual diuresis at each face-to-face visit.

**5.2. Drugs: storage and administration**

Isoniazid/pyridoxine hydrochloride (Cemidon®), rifampicin (Rifaldin® or Rimactán®), and rifapentine (Priftin®), will be stored in a cool, dry place. Each batch of tablets will be labelled in the Pharmacy Department and then transported to the outpatient clinic where participants will be attended to. PI in each centre will record the batch number and its expiration date, to warrant sample traceability as RD 1090/2015 establishes, with the rest of data required.

**5.3. Medications permitted and not permitted during the trial**

- **Medications not permitted with isoniazid.** The following medications must not be given concomitantly with isoniazid: carbamazepine and disulfiram. Although, according to the datasheet of the Agencia Española del Medicamento y Productos Sanitarios (AEMPS) *(attachment 1a)*, these are relative contraindications, for the purpose of the study, these two drugs will not be permitted concomitantly with isoniazid.
- **Medications not permitted with rifampicin.** The following medications must not be given concomitantly with rifampicin, as they are contraindicated in the datasheet of the drug, because of major interaction *(attachments 1b & 1c)*: antiretrovirals (saquinavir/ritonavir o elvitegravir/cobicistat, atazanavir, darunavir, fosamprenavir, tipranavir, rilpivirine o dolutegravir/rilpivirine), antihypertensive drugs (nifedipine, nimodipine, nisoldipine o nitrendipine), protease inhibitors anti-C virus hepatitis (glecaprevir/pibrentasvir o elbasvir/grazoprevir), antifungal drugs (ketoconazole, itraconazole y voriconazole), immunosuppressors (cyclosporin, tacrolimus), antimalarials (artemether/lumefantrine).

For other medications with significant interaction with rifampicin, they can be given if under the investigator’s judgement, with dose adjustment and close monitoring of the potential derived adverse events, the benefits outweigh the potential harms.

- **Medications not permitted with rifapentine.** Medications with significant interaction with rifapentine can be given if under the investigator’s judgement, with dose adjustment and close monitoring of the potential derived adverse events, the benefits outweigh the potential harms *(attachment 1d)*.

**5.4. Discontinuation of study drugs due to adverse events**

Certain events or conditions may necessitate temporary discontinuation of the study medications. Patients who experience such events or conditions, however, will still be "on study" and will be followed until study completion. Any patient for whom the study medication is temporarily discontinued will be restarted on study medication as soon as possible. If study drugs are definitively discontinued, alternative therapy for latent TB may be administered at the investigator's discretion.

- **Temporary Discontinuation**

Criteria for temporary discontinuation of the study medication:

- Development of a toxicity that, depending on its nature and severity, requires discontinuation of the study medication until the toxicity resolves as indicated in the preceding toxicity management section.
- Development of another medical condition that makes the administration of the study drug inadvisable. The decision to temporarily discontinue the study medication in this situation will be at the investigator's discretion. The period during which the patient is off study medication will be as short as clinically possible.
- **Definitive Discontinuation**

Criteria for definitive discontinuation of study therapy:

- Development of a toxicity that warrants definitive discontinuation of any study medications.
- The patient refuses further study therapy. If a patient refuses further study medication, they will be treated with a non-study regimen, and there will only be a post-treatment visit, four weeks after discontinuing the treatment.
- In the investigator's judgment, continuation of the medication is no longer in the best interest of the patient.
- Liver toxicity: ALT and/or AST elevation >5-fold of the upper limit of the normality or clinical hepatitis during treatment for latent TB infection with one of the IMPs.
- Neurotoxicity: Encephalopathy, dizziness, or cerebellar syndrome during treatment with INH-including regimens. It is commonly referred to as feeling “drunk”. It is usually invalidating and improved after discontinuing treatment.

**6. ASSESSMENT OF EFFICACY**

**6.1. Efficacy parameters**

The outcome to assess efficacy will be completion of the assigned treatment.

**6.2. Methods and timing for assessment**

Assessment of treatment completion will be done at the end of the maximum pre-specified time frame in which the treatment should be completed (see **section 3.1. “Statement of the endpoints”** for definition of treatment completion). The time frame will be different for each of the three study arms: 16 weeks since the date of the first treatment dose, for the 3HR arm (control); 14 weeks since the date of the first treatment dose, for the 3HP arm (experimental 1), and 20 weeks since the date of treatment dose, for 4R arm (experimental 2).

**7. ASSESSMENT OF SAFETY**

**7.1. Monitoring, recording, and reporting of adverse events**

**Adverse event (AE)** is any noxious, unintended, or untoward medical occurrence that may appear or worsen in a subject during the study. It may be a new intercurrent illness, a worsening concomitant illness, an injury, or any concomitant impairment of the subject’s health, including laboratory test values, regardless of aetiology. Any worsening (i.e., any clinically significant adverse change in the frequency or intensity of a pre-existing condition) should be considered an AE.

Any sequel of an accidental or intentional overdose of an investigational product should be reported as an AE or serious adverse event (SAE). In the event of overdose, the subject should be monitored as appropriate and should receive supportive measures as necessary. All subjects will be monitored for AEs during the study.

**All AEs** will be recorded by the investigator from the date the subject gives the written consent to four weeks after finishing the assigned treatment, as well as those serious adverse events made known to the Investigator at any time thereafter that are suspected of being related to one of the Investigational Medical Products (IMPs).

**All AEs** and **SAEs** will be recorded on the AE page of the CRF and in the subject’s source documents.

**7.2. Reference safety information**

In this study the reference safety information (RSI) will be the Summaries of Products Characteristics of IMPs:

- Isoniazid/pyridoxine hydrochloride (Cemidon®)
- Rifampicin (Rifaldin® or Rimactán®)
- Rifapentine (Priftin®)

**7.3. Evaluation of adverse events**

A qualified Investigator will evaluate all adverse events as to:

- **Seriousness**
- **Serious adverse event.** A SAE is any AE occurring at any dose that:
- Results in death
  - Is life-threatening (i.e., in the opinion of the Investigator, the subject is at immediate risk of death from the AE)
  - Requires participant inpatient hospitalization or prolongation of existing hospitalization (hospitalization is defined as an inpatient admission, regardless of length of stay)
  - Results in persistent or significant disability/incapacity (a substantial disruption of the subject’s ability to conduct normal life functions)
  - Results in a congenital anomaly/birth defect
  - Important medical events are defined as those occurrences that may not be immediately life threatening or result in death, hospitalization, or disability, but may jeopardize the subject or require medical or surgical intervention to prevent one of the other outcomes listed above
  - **Events not considered to be SAEs are hospitalizations for:**
- A standard procedure for protocol therapy administration. However, hospitalization or prolonged hospitalization for a complication of therapy administration will be reported as an SAE.
- Routine treatment or monitoring of the studied indication not associated with any deterioration in condition
- The administration of blood or platelet transfusion as routine treatment of studied indication. However, hospitalization or prolonged hospitalization for a complication of such transfusion remains a reportable SAE.
- A procedure for protocol/disease-related investigations (e.g., surgery, scans, endoscopy, sampling for laboratory tests, bone marrow sampling). However, hospitalization or prolonged hospitalization for a complication of such procedures remains a reportable SAE.
- Hospitalization or prolongation of hospitalization for technical, practical, or social reasons, in absence of an AE
- A procedure that is planned (i.e., planned prior to starting of treatment on study); must be documented in the source document and the CRF. Hospitalization or prolonged hospitalization for a complication remains a reportable SAE.
- An elective treatment of a pre-existing condition unrelated to the studied indication
- Emergency outpatient treatment or observation that does not result in admission, unless fulfilling other seriousness criteria above
- If an AE is considered serious, both the AE page of the CRF and the SAE Report Form must be completed. For each AE/SAE, the Investigator will provide information on severity, and start and stop dates, relationship to IMP, action taken regarding IMP, and outcome.
- **Casualty**

The Investigator must determine the relationship between the administration of IMP and the occurrence of an AE/SAE as Not Suspected or Suspected as defined below:

**Not related**: A causal relationship of the adverse event to IMP administration is unlikely or remote, or other medications, therapeutic interventions, or underlying conditions provide a sufficient explanation for the observed event.

**Related:** There is a reasonable possibility that the administration of IMP caused the adverse event. ‘Reasonable possibility’ means there is evidence to suggest a causal relationship between the IMP and the adverse event.

Causality should be assessed and provided for every AE/SAE based on currently available information. Causality is to be reassessed and provided as additional information becomes available.

- **Unexpectedness**

An unexpected adverse reaction (UAR) is defined as any adverse reaction whose nature or severity is not consistent with the reference safety information (RSI).

A **suspected unexpected serious adverse reaction (SUSAR)** is an adverse reaction that is both serious and unexpected.

- **Duration**

For both AEs and SAEs, the Investigator will provide a record of the start and stop dates of the event.

- **Action taken with IMP and/or adverse event**

The Investigator will report the action taken with IMP because of an AE or SAE, as applicable (e.g., discontinuation or reduction of IMP, as appropriate) and report if concomitant and/or additional treatments were given for the event.

- **Outcome**

The investigator will report the outcome of the event for both AEs and SAEs.

All SAEs that have not been resolved upon discontinuation of the subject’s participation in the study must be followed until recovered, recovered with sequelae, not recovered (death due to another cause) or death (due to the SAE).

**7.4. Abnormal laboratory values**

An abnormal laboratory value is an AE if the abnormality: results in discontinuation from the study; requires treatment, modification/interruption of IMP dose, or any other therapeutic intervention; or is judged to be of significant clinical importance.

Regardless of severity grade, only laboratory abnormalities that fulfill a seriousness criterion need to be documented as a serious adverse event.

If a laboratory abnormality is one component of a diagnosis or syndrome, then only the diagnosis or syndrome should be recorded on the AE page of the CRF. If the abnormality was not a part of a diagnosis or syndrome, then the laboratory abnormality should be recorded as the AE. If possible, the laboratory abnormality should be recorded as a medical term and not simply as an abnormal laboratory result (eg, record thrombocytopenia rather than decreased platelets).

**7.5. Pregnancy**

The Investigator should report all pregnancies in female clinical trial subjects or study subject’s partner to UICEC-IDIBELL within 24 hours of becoming aware of the information, using the UICEC-IDIBELL Pregnancy Notification Form.

- **Female of childbearing potential**

Pregnancies and suspected pregnancies (including elevated βhCG or positive pregnancy test in a female subject of childbearing potential regardless of age or disease state) occurring while the subject is on IMP, or within the time considered in the RSI of the subject’s last dose of IMP, are considered immediately reportable events. If the IMP is rifapentine, it must be discontinued immediately. The pregnancy/ suspected pregnancy must be reported to UICEC-IDIBELL by email and by sending a completed Pregnancy Notification Form. The female subject may be referred to an obstetrician-gynaecologist or another appropriate healthcare professional for further evaluation

The Investigator will follow the female subject until completion of the pregnancy, and must notify UICEC-IDIBELL immediately about the outcome (either normal or abnormal outcome) of the pregnancy (including spontaneous abortion or voluntary termination, details of birth and the presence or absence of any birth defects, congenital abnormalities or maternal and new-born complications) by email, and by sending a completed Pregnancy Notification Form.

If the outcome of the pregnancy was abnormal (e.g., spontaneous, or therapeutic abortion), the Investigator should report the abnormal outcome as an AE. If the abnormal outcome meets any of the serious criteria, it must be reported as an SAE to UICEC within 24 hours of the Investigator’s knowledge of the event using the SAE Notification Form.

All neonatal deaths that occur within 28 days of birth should be reported, without regard to causality, as SAEs. In addition, any infant death within 28 days that the Investigator suspects is related to the in-utero exposure to the IMP should also be reported to UICEC-IDIBELL as an SAE within 24 hours of the Investigator’s knowledge of the event using the SAE Notification Form. To ensure that the investigator will perform the follow-up, if UICEC-IDIBELL has not received the follow-up information, UICEC-IDIBELL will contact the investigator two (2) weeks after expected delivery date for this accomplishment. Every infant must be followed for 28 days after delivery (if applicable).

- **Male subjects**

If a female partner of a male subject taking investigational product becomes pregnant, the male subject taking IMP should notify the Investigator, and the pregnant female partner should be advised to call their healthcare provider immediately.

If a pregnancy related event is reported in a female partner of a male subject, the investigator should ask if the female partner is willing to share information with UICEC-IDIBELL and allow the pregnancy related event to be followed up to completion.

**7.6. Reporting of serious adverse events**

All **SAEs** (initial and follow up information) must be reported to UICEC-IDIBELL within 24 hours of the Investigator’s knowledge of the event by email, using the SAE Report Form and the contact information provided in the protocol. This requirement applies to all SAEs, regardless of relationship to IMP that occur during the study or any SAEs made known to the Investigator at any time thereafter that are suspected of being related to IMP.

The SAE report should provide a detailed description of the SAE and include a concise summary of hospital records with relevant items or other relevant documents. If a subject died and an autopsy has been performed, copies of the autopsy report and death certificate are to be sent to UICEC-IDIBELL if available. UICEC-IDIBELL will confirm that the notification is valid within days by e-mail. If the notification is not valid, UICEC-IDIBELL will request the information to the researcher via email.

**7.7. Procedure for expedited reporting of serious unexpected adverse reactions**

If an adverse event report meets the following three criteria, then the event is reportable in an expedited manner to all concerned Regulatory Authorities, concerned ethical committee and to all participating investigators:

- Serious
- Unexpected
- Associated with the use of some Investigational Medicinal Products.

All relevant information about suspected serious unexpected adverse reactions that are fatal or life-threatening is reported as soon as possible and, in any case, no later than seven calendar days after the investigator is aware of by UICEC-IDIBELL of such a case, and that relevant follow-up information is subsequently communicated within an additional eight calendar days.

All other SUSARs will be reported as soon as possible but within a maximum of 15 calendar days of knowledge. The Sponsor will notify regulatory authorities, as soon as possible and no later than 15 days after having knowledge of it, any information that could alter the benefit/risk relationship of the investigational medicinal product (e.g. an increase in the rate of occurrence of the expected SAR, SUSARs that occur after the completion of a clinical trial, new events related to the conduct of the trial or the development of the investigational medicinal product, any recommendation of the Data Monitoring Committee where relevant for the safety of subjects, etc.).

**7.8. Special interest adverse events**

- Liver toxicity
- Neurotoxicity
- Hypersensitivity

These events will be reported as a SAE, they will be considered an “Important Medical Event” even if no other serious criteria apply.

**7.9. Annual safety report**

During the study, the sponsor or a delegated unit by the sponsor will prepare periodic safety reports annually following the recommendations outlined in the ICH E2F guidelines, and they must be submitted to the regulatory authorities and the ECs involved following the timetable established in the legislation.

**7.10. Report to the investigators**

The sponsor shall present the safety information that could affect the safety of the participants included in the study to the investigators as soon as possible.

The SUSAR information will be sent annually through a line listing, together with a summary of the data analysis. In addition, the investigator will be informed throughout the study about all safety aspects, including amendments to the protocol due to safety reasons.

**7.11. Data and safety monitoring board** **(DSMB)**

The DSMB will meet once the first 110 participants have been recruited, and there are at least 6 months of follow-up for the last participant is included.

The membership, and the study aspects to be reviewed, will be specified in the DSMB Charter. The DSMB chairperson will be responsible for providing a written report of findings and recommendations to the sponsor in a timely manner. The sponsor will be responsible for informing the study sites and appropriate regulatory authorities of any DSMB recommendation relating to conduct of the study.

**8. STATISTICAL METHODS**

**8.1. Statistical analysis**

The efficacy analysis will be performed on the modified intention-to-treat population, which will consist of all randomized subjects. The proportions of participants who completed the assigned treatment in the 3HR arm (control) will be compared with the proportions in either 3HP arm (experimental 1) or 4R arm (experimental 2). Since renal replacement therapy could have a potential influence on medication tolerance, especially INH, and thus could influence outcome, the analysis for the primary endpoint will be performed for the population as a whole and by subgroups (non-dialysis, on haemodialysis, or on peritoneal dialysis).

For safety, analyses will be performed on all randomized subjects who have received at least one dose of the assigned study treatment. Likewise, proportions of permanent discontinuation of the assigned treatment, related and unrelated to the study medication, and death, in the control arm will be compared with either experimental arm.

Statistical analyses will be performed by two investigators of the Biostatistics Unit of the IDIBELL, who will remain unaware of the trial-group assignments until the analyses have been completed. Differences between arms will be tested by Student’s t-test, if normally distributed, or a Wilcoxon two-sample test, if not normally distributed, or by chi-square analysis for categorical variables. Whenever possible, estimators will be given 95%CI. Statistical significance was set at a p-value <0.025. Treatment of the data and analysis will be performed with the statistical package R, version 3.4.1 or superior.

**8.2. Sample size**

The sample size has been calculated to demonstrate a clinically significant better completion with the 3HP or 4R regimens than the 3HR regimen. With an α= 0.025, β= 0.20, and 5% expected losses, and assuming a 0.75 proportion of treatment completion in the control arm (3HR), according to our previous experience, 225 subjects (75 per arm) will be needed to demonstrate, if exists, a 0.16 increase in treatment completion rates in the experimental arms (4R or 3HP) respectively. Sample size was calculated with GIGAcalculator (<https://www.gigacalculator.com/calculators/power-sample-size-calculator.php>).

**9. ETHICS**

The Investigator will ensure that this study is conducted in accordance with the principles of the Declaration of Helsinki, ICH Guidelines for Good Clinical Practice and RD 1090/2015), and in full conformity with relevant regulations

The protocol, informed consent form (ICF), participant information sheet (PIS) and any applicable documents will be submitted to an appropriate Ethics Committee (EC) and Regulatory Authority (AEMPS) for written approval. All substantial amendments to the original approved documents will be also sent to an appropriate Ethics Committee (EC) and Regulatory Authority (AEMPS) for written approval.

**9.1. Informed consent**

The participant must personally sign and date the latest approved version of the informed consent form before any study-specific procedures are performed.

Written and verbal versions of the Participant Information Sheet (PIS) and Informed Consent Form (ICF) will be presented to the participants detailing no less than: the exact nature of the study; the implications and constraints of the protocol; the known side effects and any risks involved in taking part. It will be clearly stated that the participant is free to withdraw from the study at any time for any reason without prejudice to future care, and with no obligation to give the reason for withdrawal.

The participant will be allowed as much time as wished to consider the information, and the opportunity to question the Investigator or other independent parties to decide whether they will participate in the study. Written Informed Consent will then be obtained by means of participant dated signature and dated signature of the person who presented and obtained the informed consent. The person who obtained the consent must be suitably qualified and experienced and have been authorized to do so by the Principal Investigator. For its obtention, the responsible investigators will comply with the current legislation (RD 1090/2015) and ethical principles of the Helsinki declaration. A copy of the signed ICF will be given to the participants. The original signed form will be retained at the study site *(attachment 3)*.

**9.2. Subjects’ confidentiality**

Treatment, communication, and cession of personal data of all the participants in the study will be guided by the Organic Law 3/2018 of 5 December on the Protection of Personal Data and Guarantee of Digital Rights (LOPDPGDD)." Data collected for the study will be identified by a code, and only the authorized investigators will be able to relate the participant's data with their names and clinical identification code.

**10. FUNDING AND INSURANCE**

- **Funding.** The trial is funded with a grant of 149,435€ through a competitive call, by the Instituto de Salud Carlos III of the Ministry of Science and Innovation of the Spanish Government, (Acciones Estratégicas de Salud 2021. Grant number: PI21/004444).
- **Insurance.** Insurance will be contracted to cover the civil liability that may arise from the sponsor and the investigator for the damages caused to the participants. The available budget for the insurance is 15,000€.

**11. AGREEMENT TO PUBLISH**

Miguel Santin MD, PhD, as Sponsor of the trial, agrees to make publicly available the main study results, regardless of their outcome.

**12. ATTACHMENTS**

**Attachment 1a.** Data sheet of Cemidon®

**Attachment 1b.** Data sheet of Rifaldin®

**Attachment 1c.** Data sheet of Rimactán®

**Attachment 1c.** Data sheet of Priftin®

**Attachment 2.** Scheduled assessment visits

**Attachment 3a.** Informed consent form

**Attachment 3b.** Appendix
